# Molecular Characterisation of *Fusarium* Species Causing Common Bean Root Rot in Uganda

**DOI:** 10.3390/jof11040283

**Published:** 2025-04-03

**Authors:** Samuel Erima, Moses Nyine, Richard Edema, Allan Nkuboye, Nalule Habiba, Agnes Candiru, Pamela Paparu

**Affiliations:** 1 National Agricultural Research Organization, National Crops Resources Research Institute, Namulonge, Kampala P.O. Box 7084, Uganda; kayongoallan@gmail.com (A.N.); candiruagnes95@gmail.com (A.C.); pamela.paparu@gmail.com (P.P.); 2 Department of Crop Science and Horticulture, School of Agricultural Sciences, Makerere University, Kampala P.O. Box 7062, Uganda; redema14@gmail.com (R.E.); hnalule814@gmail.com (N.H.); 3 Faculty of Agriculture and Environmental Sciences, Muni University, Arua P.O. Box 725, Uganda; 4 Platain Breeding Program, International Institute of Tropical Agriculture (IITA), PMB 5320, Oyo Road, Ibadan 200001, Oyo State, Nigeria; m.nyine@cgiar.org

**Keywords:** common bean, dry beans, fusarium root rot, genetic diversity

## Abstract

Recently, Fusarium root rot (FRR)-like symptoms were observed in Uganda’s agroecology zones, prompting the National Agricultural Organisation (NARO) to conduct a disease survey. The survey reports indicated FRR as the second most prevalent root rot disease of common bean in Uganda after Southern blight. Ninety nine *Fusarium* spp. strains were obtained from samples collected during the surveys. The strains were morphologically and pathogenically characterised and confirmed to cause Fusarium root rot as observed in the field. However, molecular characterization of the strains was not conducted. In this study, therefore, 80 of the strains were characterized using partial sequences of translation elongation factor 1-alpha (TEF-1α) gene, beta tubulin (β tubulin) gene and internal transcribed spacers (ITS) region of ribosomal RNA to determine species diversity. High-quality Sanger sequences from the target genes were compared to the sequences from *Fusarium* species available in the National Centre for Biotechnology Information coding sequences (NCBI-CDS) database to determine the most likely species the strains belonged. The sequences from our strains were deposited into the NCBI gene bank under ID#288420, 2883276, 2873058 for TEF-1α, β tubulin and ITS respectively. The *Fusarium* species identified included; *F. oxysporum*, *F. solani*, *F. equiseti F. delphinoides*, *F. commune*, *F. subflagellisporum*, *F. fabacearum*, *F. falciforme*, *F. brevicaudatum*, *F. serpentimum*, *F. fredkrugeri* and *F. brachygibbosum*. The diversity of these *Fusarium* species needs to be taken into consideration when developing breeding programs for management of the disease since currently there is no variety of common bean resistant to FRR in Uganda.

## 1. Introduction

The common bean (*Phaseolus vulgaris* L.) is the most widely distributed *Phaseolus* species grown all over Africa [1]. According to FAO [2], Uganda produced 1,008,410 tons of common bean in 2016, making it the second largest producer after Tanzania at 1,200,000 tons. The production in Uganda is mostly done by small-scale farmers with land holdings of between 0.1 and 4 hectares [3]. Common bean production faces several constraints. Among the biotic constraints, fungal root rots are key [4,5]. Many fungal pathogens such as *Sclerotium rolfsii*, *Fusarium* species, *Pythium* species, *Macrophomina phaseolina* and *Rhizoctonia solani* have been reported to cause bean root rot [6,7,8].

Fusarium root rot disease of common bean, hereafter referred to as FRR, was reported as the second most important bean root rot disease in Uganda after Southern blight caused by *Sclerotium rolfsii* Sacc. (teleomorph *Arthelia rolfsii* (Curzi) C. C. Tu and Kimbr.) [9]. Several different species of Fusarium have been reported to cause FRR in common bean in several countries. *Fusarium cuneirostrum* from *Fusaium solani* species complex was reported to cause FRR in several countries such as the China, United States, Brazil, Canada and Uganda [10,11,12]. Other Fusarium species in the *Fusarium solani* species complex that have been reported to cause Fusarium bean root rot include *Fusarium equiseti*, *Fusarium graminearum*, *Fusarium rodelens* and *Fusarium sporochoides* [13,14]. Meanwhile, Fusarium oxysporum had been documented to cause vascular wilts, however, it has been found to cause Fusarium root rot in common bean [13,15,16].

The symptoms of FRR include longitudinal reddish-brown lesions on hypocotyls accompanied by longitudinal fissures or cracks with dying root tissues turning reddish brown. Infected plants are chlorotic, beginning with the primary leaves, stunted, and plants may wilt completely or undergo premature senescence. Yield losses due to Fusarium root rot (FRR) have been reported to reach 86% in severely affected soils [17]. The legumes program of National Agricultural Research Organisation conducted a survey of seven agroecological zones that included the South Western Highland (SWH), Western Mixed Farming system (WMFS), Lake Victoria Crescent and Mbale Farmlands (LVC), Eastern Highlands (EH), Northern Mixed Farming System (NMFS), North Eastern Dry Lands (NEDL) and West Nile Mixed Farming System (WNMFS). During these surveys, wilting plants with Fusarium root rot like symptoms were collected and used for pathogen isolation. *Fusarium* species strains were obtained and characterised both morphologically in culture media and phenotypically through pathogenicity studies [18]. While understanding the molecular diversity among pathogen populations to facilitate the development of host plant resistance is important, genetic diversity studies were not conducted in the earlier study.

Genetic diversity among *Fusarium* species has been studied using DNA-based markers such as Inter Simple Sequence Repeats (ISSR) and Single Sequence Repeats (SSR) [19], Amplified Fragment Length Polymorphism (AFLP) [20], Restriction Fragment Length Polymorphism (RFLP) and Randomly Amplified Polymorphic DNA (RAPD) [21]. Internal transcribed spacers region of the ribosomal RNA (ITS), beta tubulin (β tubulin) gene region [22], calmodulin gene region [23] and sequences from translation elongation factor 1 alpha (*TEF1-α*) gene have been widely used as taxonomic markers for fungal species identification [5,24,25].

Several studies have reported the effectiveness of the *TEF1-α* gene in fungal species identification, disease diagnosis and postharvest fungal toxicity surveys in crops such as coffee (*Coffea* species [26], sugar beet (*Beta vulgaris*) [27], bread wheat (*Triticum aestivum* L.) [28], millet (*Eleusine coracana* Gaertn.), sorghum (*Sorghum bicolor* L. Moench.), maize (*Zea mays* L.), groundnuts/peanuts (*Arachis hypogea* L.) and sesame (*Sesamum indicum* L.) [29]. The TEF-1α gene was used to identify and classify dermatophytes and it provided a high degree of differentiation between species that were closely related [25]. Similarly, partial sequences of the β tubulin gene region have been used to study molecular diversity and identification of *Fusarium* species. Kalman et al. [22] used the β tubulin gene region to identify *Fusarium* species causing basal rot in *Allium cepa*. Several authors have used ITS for the identification of fungal species [30]. Singha et al. [31] used ITS 1 and 4 to identify *Fusarium* species causing wilts in tomatoes and was able to detect several *Fusarium* species such as *F. oxysporum*, *F. equiseti*, *F. proliferatum.* Other authors have used other gene regions such as clamodulin (*cam*), RNA polymerase second largest subunit (rpb2) genes and the Cytochrome oxydase 1 (COX 1) gene region for identification of *Fusarium* to species level. [23,32]. Though Calmodulin primers were able to distinguish the *Fusarium* species [13], in the study by Gilmore [32] many of the species of *Fusarium* shared similar *COX* 1 partial gene sequences making *COX* 1 barcoding in *Fusarium* entirely infeasible.

The study by Paparu et al. [9] showed an increasing significance of FRR in Uganda’s agroecology zones. The disease was the second most prevalent after Southern blight. However, there is limited information on the diversity of *Fusarium* species causing root rot in common bean. To fill the observed knowledge gap, we sought to identify *Fusarium* species causing Fusarium root rot in Uganda. This information is useful in the development of host plant resistance, which is a key disease management strategy for smallholder farmers in sub-Saharan Africa.

## 2. Materials and Methods

### 2.1. Origin of Fusarium Species Strains Used

A collection of 99 hyphal tipped *Fusarium* species strains previously stored on filter papers originated from 6 agroecological zones of Uganda. These included, South Western highland (SWH), Western mixed farming system (WMFS), Lake Victoria Crescent and Mbale farmlands (LVC), Eastern highlands (EH), Northern mixed farming system (NMFS) and North Eastern dry lands (NEDL). The Strains were reactivated by growing them on Potato Dextrose Agar (PDA, Esvee Biologicals, Mumbai, India) media (39 g PDA in 1 L distilled water) for 14 days. Strains with growth rates of less than 0.6 cm per day were selected since *Fusarium* species that cause root rot on common bean were reported to have low growth rates [33].

### 2.2. DNA Extraction from Fusarium Species Strains

DNA was extracted from two-week-old mycelia of the 99 previously mentioned strains using a modified Cetyl trimethylammonium bromide (CTAB) protocol previously used by the Joint Research Council (JRC), European commission [34]. Actively growing mycelia were harvested by scraping them off the surface of the PDA into sterile Petri dishes. The mycelia were oven-dried over night at 30 °C. About 0.02 g of the mycelia was loaded into 2 mL Eppendorf tubes containing beads. The mycelia were ground into a fine powder using an automated tissue homogenizer and cell lyser Geno Grinder (1600 MiniG, Cole-Parmer, Chicago, IL, USA) for 3 min at 1450 revolutions per minute (rpm). Seven hundred microliters (700 μL) of DNA extraction buffer (2% CTAB, 50 mM EDTA pH 8.0, 100 mM Tris-Base pH 8.0, 2% PVP-40, 1% NaSO_3_, 1.4 M NaCl and 1% beta 2-mercaptoethanol) was added and the mycelia homogenized for another 2 min in the Geno grinder. Samples were incubated at 65 °C for 30 min with occasional shaking. Tubes were then centrifuged at 12,000 revolutions per minute for 10 min. Five hundred microliters (500 μL) of the supernatant was picked and transferred into new 2-mL Eppendorf tubes. Four hundred and fifty microliters (450 μL) of chloroform and iso amyl alcohol at the ratio of 24:1 was added to each sample, and the tubes were shaken for 2 min. Samples were then centrifuged at 10,000 strokes per minute for 10 min. Four hundred microliters (400 μL) of supernatant containing DNA was transferred into well-labeled 1.5 mL Eppendorf tubes. Four hundred and fifty microliters (450 μL) of Isopropanol (stored at −20 °C) and 40 µL of 3 M Sodium Acetate solution were added to the DNA and incubated at −20 °C for 2 h to precipitate the DNA. The tubes were then centrifuged at 15,000 rpm for 15 min to separate the DNA from the Isopropanol. The supernatant was decanted and the pellet washed with 500 µL of 70% ethanol by centrifuging at 7000 rpm for 10 min. The supernatant was decanted and DNA pellets air dried for 1 h at room temperature (25–30 °C). DNA pellets were then resuspended in 100 µL of elution buffer, the DNA concentration was assessed using a NanoDrop (ND-1000) (Thermo Fisher, Waltham, MA, USA), and it was stored at −80 °C. Generally, the concentration of all the samples was above 500 ng/μL, while the A260/A280 ratios ranged between 1.9 to 2.1.

### 2.3. Fusarium Species Identification Using TEF1-α, β Tubulin and ITS Partial Sequences

A multi-gene approach was used to identify the 99 *Fusarium* species strains. The primer sequences used to amplify portions of the target genes included *TEF1-α* gene forward primer (Ef 1: 5′-ATGGGTAAGGARGACAAGAC-3′) and reverse primer (Ff 2: 5′-GGARGTACCAGTSATCATGTT-3′) [12]. ITS forward primer ITS 1 (GGAAGTAAAAGTCGTAACAAGG) and reverse primer ITS 4 (TCCTCCGCTTATTGATATGC) were used for the amplification of the ITS region of ribosomal RNA [35]. Meanwhile, the β tubulin gene region was amplified using a forward primer (T1-AACATGCGTGAGATTGTAAGT) and reverse primer (T2-TAGTGACCCTTGGCCCAGTTG) [22].

A PCR master mix (Bioneer Corporation, Daejeon, Republic of Korea) was used in the amplification reactions according to the manufacturer’s instructions. A total reaction volume of 30 µL was used and it consisted of 15 µL premix, 1 µL of each reverse and primer, 3 µL of DNA and 10 µL of DNase free water. The PCR conditions included an initial denaturation at 95 °C for 5 min followed by 35 cycles of denaturation at 95 °C for 3 min, annealing at the various annealing temperature for the respective primers for 40 s, extension at 72 °C for 1 min and a final extension at 72 °C for 5 min. The annealing temperature for each primer was as follows: *TEF1-α* at 55 °C, ITS at 53 °C and β tubulin at 57 °C. For quality control, 5 µL of PCR products from each sample were electrophoresed alongside the 100 bp DNA ladder in a 1.5% agarose gel containing Gel-red fluorescent dye (Botium) in 1x TBE buffer at 100 V for 40 min. Gels were documented using a bench top Transilluminator (BioDoc-It Imaging System 8. Cole-Parmer, Chicago, IL, USA). However, out of the 99 strains, 80 of the strains were able to show amplification with the various primers. These PCR products from the 80 strains were purified using the AccPrep^TM^ purification kit (Bioneer Cooperation, Daejeon, Republic of Korea) following the manufacturer’s instructions. The products were sequenced using their respective reverse primers in an ABI13730XL Sanger sequencing machine (Applied Biosystems, Waltham, MA, USA) using the BigDye Terminator v3.1 sequencing kit (Applied biosystems, USA) at Macrogen (Amsterdam, The Netherlands).

### 2.4. Growth Rate, Disease Severity Index (DSI) and Morphological Characteristics of Fusarium Species Strains

The average disease severity index (DSI) and growth rate of strains was obtained from Erima et al. [18]. In the study by Erima et al., the inoculum was prepared by cutting 1-cm^2^ agar plugs from 2-week-old cultures on PDA and inoculating them in 50 g of sterile millet in an autoclave bag. Spore concentration could not be used to measure the inoculum because some of the isolates did not produce conidia. Bags were incubated at 25 °C for two weeks until mycelia had fully covered the millet. Wooden trays of 100 cm × 35 cm × 10 cm were used to set up the experiment in the greenhouse. Ten grams of the inoculum was mixed with about 20 kg of soil in the wooden trays. Then, 16 seeds of each of the five test lines were planted in each tray with a replicate. A control tray which was un-inoculated was also planted with the test varieties. Virulence was then assessed at 28 days after planting using a scale of 1 to 9.

Meanwhile, the growth rate was determined on PDA using Petri dishes with a 9-cm diameter. A cross was made on the bottom of the Petri dish to mark its center. Inoculum was picked from 2-week-old cultures by tapping the mycelia with a needle. The inoculum was then transferred to the center of the marked Petri dish. Each strain was replicated three times. Growth data were collected 2 days post-inoculation by using a 30-cm ruler to measure the diameter of the colony until day 8 when mycelia for some isolates had reached the edge of the Petri dish. Information on colony color was also recorded. Microscopy was then conducted using 2-week-old cultures on PDA at 40× for selected strains of the different species. The shape and sizes of the macro- and micro-conidia were recorded, and photos were taken of the different strains.

### 2.5. Data Analysis

Sanger sequences were imported into chromas software (Chromas 2.6) for quality assessment. Low-quality bases at the 5′- and 3′-ends were trimmed off, and high-quality sequences exported as a FASTA file. The high-quality reads representing 80 *Fusarium* species strains from different agro-ecology zones were obtained and used for downstream analysis. They included 60 sequences from TEF1-α, 59 sequences from β tubulin and 58 sequences from ITS. The number of sequences of various strains varied because not all primer sets amplified the genes from the same strains. To confirm the species, the strains’ sequences were compared to the coding sequences in the National Centre for Biotechnology Information coding sequences (NCBI-CDS) database using basic local alignment search tool for nucleotides (BLASTn). The sequences were analyzed for the presence of open reading frames, exons and introns. The concordance of species’ names between two independent databases as the top hit was used to assign the species’ identities to the strains. Sequences were imported into MEGA 11.0 and aligned. A phylogenetic tree was constructed using the neighbor joining method using the TEF1-α sequences since it resolved all the *Fusarium* species. Curated *Fusarium* species sequences were deposited in the NCBI database. ITS and β tubulin could not resolve some species from *Fusarium solani* and *Fusarium oxysporum* species complexes, identifying all of them as *F. solani* and *F. oxysporum*, respectively. Data on morphological characteristics such as growth rate, virulence and colony color were obtained from Erima et al. [18]. Tukey’s honestly significant difference (HSD) test was used to test the difference in virulence and growth rate between the different *Fusarium* species.

## 3. Results

### Identification of Fusarium Strains Using TEF1-α Gene, β Tubulin Gene and ITS Partial Sequences

**Figure 1:**
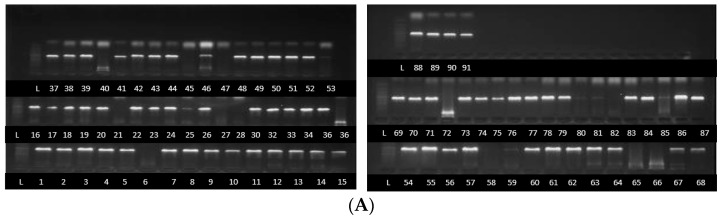

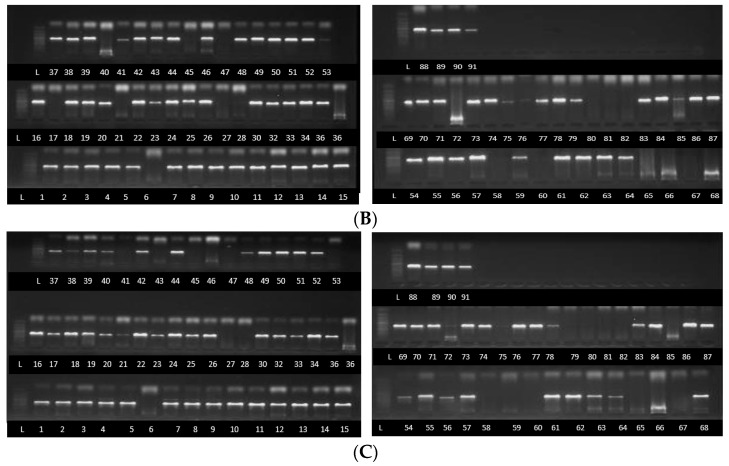
PCR product bands of *Fusarium* species strains following amplification using ITS (**A**), β tubulin (**B**) and TEF1-α primers (**C**). Some strains without bands were not detected by the primers.

Partial sequences of about 700 bp, 580 bp and 560 pb were obtained after the PCR amplification and sequencing of the PCR products of TEF1-α gene, β tubulin gene and ITS partial sequences, respectively (Figure 1). Sequences were successfully sequenced and processed for a combined total of 80 strains (TEF1-α = 60 strains, β tubulin = 59 strains and ITS = 58 strains). The sequences were deposited at the National Centre for Biotechnology Information (NCBI) under accessions PQ363745 to PQ363805, PQ497178 to PQ497237, PQ497119 to PQ497177 for ITS, TEF1-α and β tubulin, respectively.

**Figure 2:**
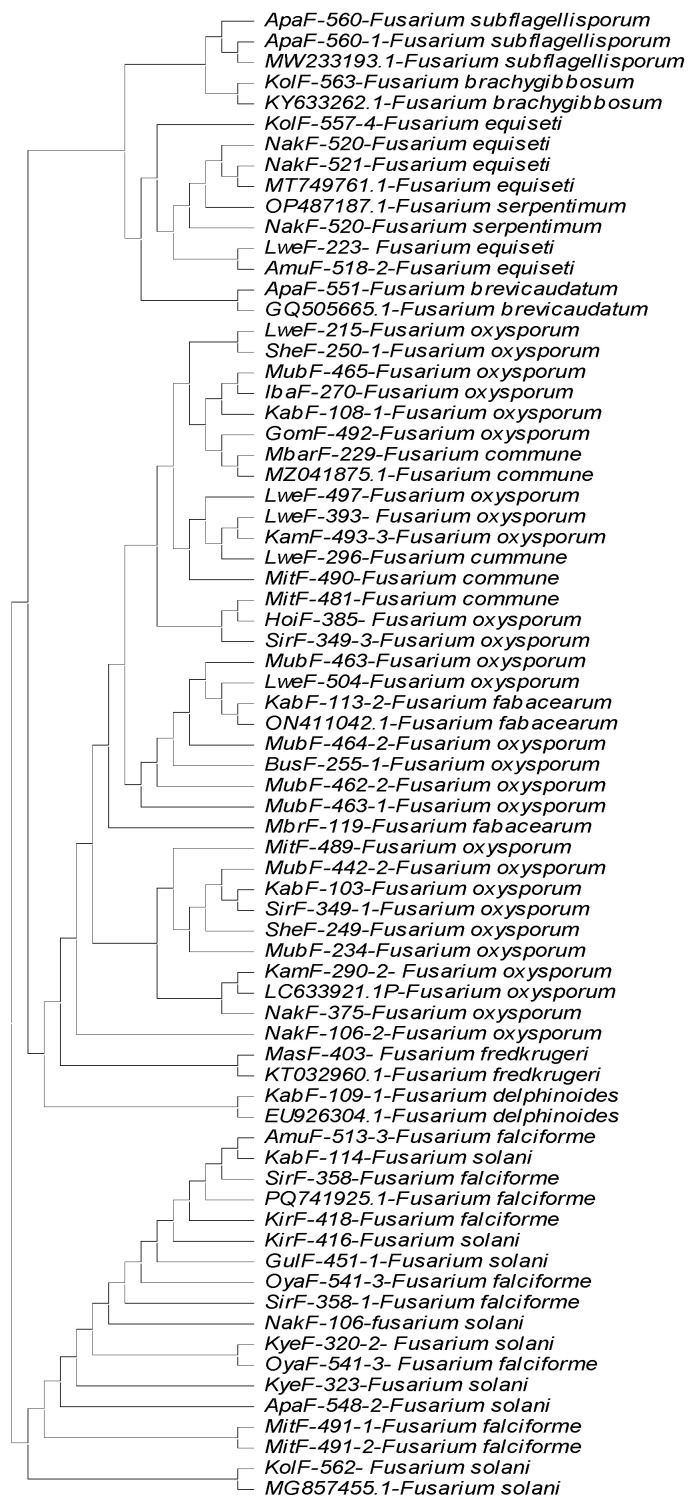
The phylogenetic tree constructed using the neighbor joining method for *Fusarium* species strains collected from six Ugandan agroecology zones. This analysis involved 72 nucleotide sequences. All ambiguous positions were removed for each sequence pair (pairwise deletion option). There were a total of 1939 positions in the final dataset. Evolutionary analyses were conducted in MEGA11.

Comparing the high-quality, trimmed Sanger sequences with the NCBI CDS database for TEF1-α, ITS and β-tubulin, we identified 12 different *Fusarium* species with sequence identities ranging from 99.9% to 100% to those of the respective reference sequences of the species in the database. Thirty seven strains were most identical to *F. oxysporum*, 13 to *F. solani*, 7 to *F. falciforme*, 9 to *F. equiseti* and 4 to *F. commune.* Meanwhile, *F. fabacearum* and *F. subflagellisporum* were each represented by two strains. Single strains of *F. delphinoides*, *F. brevicaudatum*, *F. serpentimum*, *F. fredkrugeri* and *F. brachygibbosum* were identified. A strain belonging to *Clonostachys rhizophaga* was also identified (Table 1). The TEF1-α gene had the least number of strains with missing data, and produced the longest reads with high-quality bases after trimming. However, we used the sequences from all three genes (TEF1-α, ITS and β tubulin) to dependently identify the isolates to species level (Table 1). A strain was assigned to species if at least two of the gene sequences matched with the same species with >99% identity. Due to variation in read length from ITS and β tubulin genes some strains grouped under *F. falciforme* and *F. serpentimum* by TEF1-α could not be resolved from *Fusarium solani* species complex. The two genes primers were also unable to resolve *F. fredkrugeri*, *F. commune*, *F. fabacearum*, *F. subflagellisporum* and *F. brachygibbosum* from the *Fusarium oxysporum* species complex, identifying them as *Fusarium oxysporum.* A maximum likelihood phylogenetic tree was generated using the neighbor joining method, using only the TEF1-α sequences (Figure 2). A consensus tree could not be generated by concatenating the sequences because of the high variation in length, quality and data absence of ITS and β tubulin in sequences after trimming. One strain, MitF-487-2, identified as *Clonostachys rhizophaga*, could not be included in the tree because its sequences were too divergent to align with those of the *Fusarium* species. The *Fusarium* species, their agroecology of origin and accession numbers are summarized in Table 1.

*Fusarium* species have been reported to vary in their morphological characteristics such as growth rate, virulence, and shape and sizes of microscopic structures and colony color [29,33]. The average disease severity index (DSI), growth rate and colony colors of the strains were obtained from Erima et al. [18]. The average DSI and growth rate varied among the species (Table 1 and Table S1). All the different *Fusarium* species varied significantly in disease severity index (DSI) caused to five common bean varieties (F = 3.6 *p* = 0.03). Following Tukey’s honestly significant difference (Tukey’s HSD) test, the average DSI caused by the *Fusarium* species were still significantly different from each other. *Fusarium solani* was the least pathogenic, with an average DSI of 37.2% while *F. subplagellisporum* was the most pathogenic with an average DSI of 66.6% (Table 2). The *Fusarium* species strains also varied significantly in average growth rate per day (F = 2.9 *p* < 0.001). Following Tukey’s HSD, the growth rates of *F. brachygibosum*, *F. fredkrugeri*, *F. delphinoides and F. fabacearum* were similar, while those of *F. solani*, *F. oxysporum*, *F. brevicaudatum*, *F. serpentimum*, *F. falciforme* and *F. equiseti* were also similar. The growth rates of *F. subflagellisporum* and *F. commune* were similar, while the growth rate of *C. rhizophaga* was different from that of all the *Fusarium* species strains.

**Figure 3:**
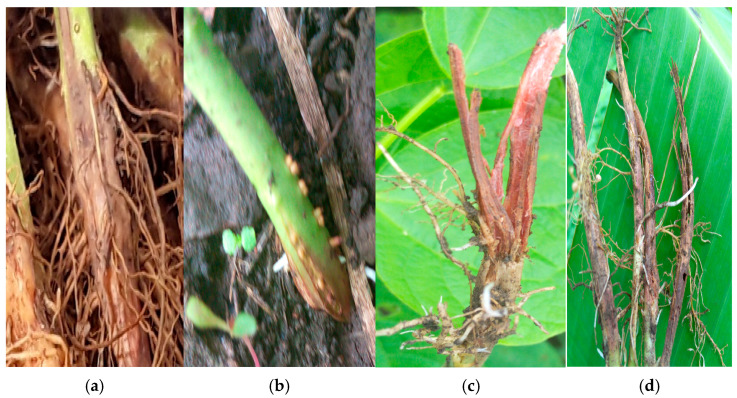
Symptoms caused by some of the strains during pathogenicity by Erima et al. [18]. (**a**) Longitudinal dark brown lesions extending along the vascular bundle past the collar of the plant, typical of *F. oxysporum*. (**b**–**d**) Longitudinal reddish brown spots with cracks and fissures leading to total reduction of the main root system.

Many of the *Fusarium* species strains exhibited multiple colors. Colony colorations such as white, white/purple, white/pink and white/cream were reported for *F. oxysporum* and *F. solani*, though specific colorations such as white/yellow and white/brown were reported for *F. solani*. All the strains of *F. equiseti* were white both on the top and bottom of the Petri dish. While *F. falciforme* and *F. commune* had strains which were white/pink, white/purple and white, the strains of *F. brevicaudatum*, *F. serpentimum*, *F. brachygibbosum*, *F. subflagellisporum*, *C. rhizophaga*, *F. delphinoides*, *F. fabacearum* and *F. fredkrugeri* were colored white, white/purple, white/purple, white/brown, white/purple, white/pink, purple and white, respectively (Figure S1, Table S1). We could not present the colony pictures of the strains of *F. brevicaudatum* and *F. serpentimum* because, after DNA extraction, the filter papers in storage were depleted. However, their colony colors were obtained from Erima et al. 2024 [18]. Photos of the symptoms caused by a few *Fusarium* species identified above were retrieved from the archives at National Crops Resources Research Institute and were observed to vary among the species. The lesions caused by *F. oxysporum* were along the vascular bundle and extended from the roots to above the soil line (Figure 3a,b), while the lesions caused by *other Fusarium* species were restricted to the root area (Figure 3c,d). Photos were not captured for every strain phenotyped, as we were not aware at that time if they belonged to different species or not.

**Figure 4:**
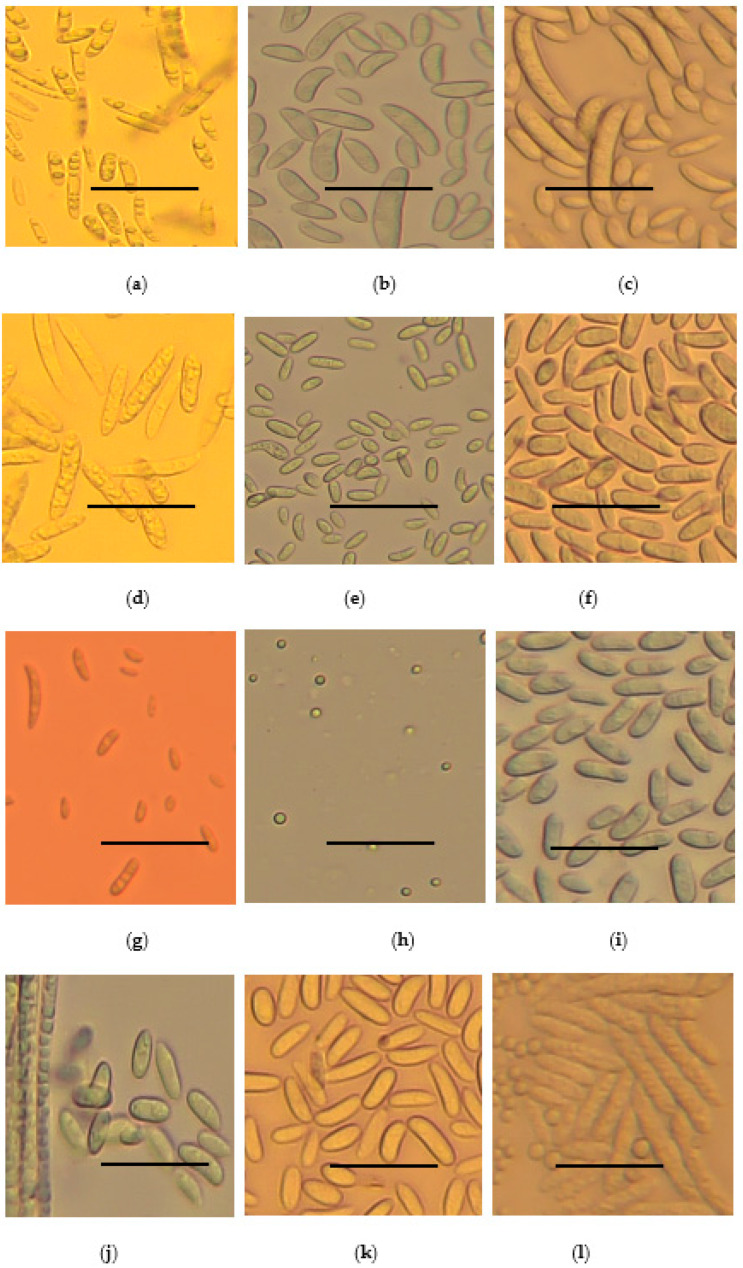
Microscopic features of *Fusarium* species strains at 40x magnification: (**a**) *F. oxysporum*, (**b**) *F. solani*, (**c**) *F. falciforme*, (**d**) *equiseti*, (**e**) *F. brachygibosum*, (**f**) *C. rhizophaga*, (**g**) *F. fredkrugeri*, (**h**) *F. subflagellisporum*, (**i**) *F. fabacearum*, (**j**) *F. delphinoides*, (**k**) *F. commune* and (**l**) *F. equiseti* macro- and micro-conidia. The scale bar in the pictures represents 100 µm.

Following microscopy, all the strains were observed to have septate hyphae. They either produced micro- or macro-conidia or both. The micro-conidia were spherical while the macro-conidia were rod-shaped, oval, or sickle-shaped. The shape and sizes of micro- and macro-conidia are summarized in Figure 4 and Table 2.

## 4. Discussion

Partial sequences of translation elongation factor 1-alpha (TEF1-α), β-tubulin, and the ITS region of ribosomal RNA were used to identify *Fusarium* species strains previously isolated from the roots of wilting common beans. *Fusarium* species identified as the main pathogens causing Fusarium root rot (FRR) in Uganda included *F. oxysporum*, *F. solani*, *F. equiseti*, *F. falciforme*, *F. flagellisporum*, *F. commune*, *F. brevicaudatum*, *F. brachyggibosum*, *F. serpentimum*, *F. frekrugeri*, *F. fabacearum* and *F. delphinoides*. The identification of *Fusarium* species based on plant disease symptoms is quite challenging. In both field and greenhouse settings, the early symptoms of FRR and wilt are similar (wilting and yellowing of leaves), and root rots sometimes occur as disease complexes. Morphological identification and classification continue to be used but with enormous challenges as it requires experienced mycologists to identify fungi to species level [36]. Despite this, the proper identification and classification of *Fusarium* spp. is important for monitoring changes in the species population and their impact on agriculture.

Previously, *Fusarium* species such as *Fusarium solani* and *Fusarium cuneirostrum* have been reported to cause bean root rot in Uganda [11,33]. Lately, many studies have taken place in other countries to identify *Fusarium* species causing common bean root rot. In China, *Fusarium* species such as *F. equiseti*, *F. oxysporum*, *F. solani* and *F. cuneirostrum* have been reported to cause common bean root rot [10,37,38,39]. The current study has identified additional *Fusarium* species such as *F. falciforme*, *F. subflagellisporum*, *F. commune*, *F. brevicaudatum*, *F. brachyggibosum*, *F. serpentimum*, *F. frekrugeri*, *F. fabacearum* and *F. delphinoides* as the causal agents of Fusarium bean root rot in Uganda.

Several members of the *Fusarium* species complex have a wide host range and diverse ecological niches, yet they also differ in their characteristics. For example, *F. equiseti* was reported to cause seedling wilting, root tip discoloration and necrosis in sugar beet by Khan et al. [40]. *Fusarium falciforme* was also reported to cause root rot in *Weigelia florida* in China [29]. According to Trabelsi et al. [41], both *F. oxysporum* and *F. brachygibbisum* cause die back in olive, trees and the two species clustered closely in the current study. The other species that clustered closely with *Fusarium oxysporum* included *F. commune*, *F. frekrugeri*, *F. subflagellisporum*, *F. delphinoides*, *F. fabacearum*, *F. brachygibbosum* and *F. brevicaudatum.* Namasaka [42] reported *F. equiseti* as a causal agent of cowpea root rot. Interestingly, the current study confirmed the species as a causal agent of common bean root rot in Uganda. Marcelo et al. [43] recovered *F*. *frekrugeri* from soil under *Musa acuminata* from Kruger national park in South Africa in undisturbed forest soil. In this study, *F*. *frekrugeri* caused a DSI of 40.3%. Meanwhile, the *F. delphinoides* strain GPK was reported to be pathogenic to chickpeas and pigeon peas by Guruprasad et al. [44].

There are at least 20 species complexes in the genus *Fusarium*. Chehri et al. [45] and Coleman [46] reported *F. falciforme* as a species under the *F. solani* species complex. In the current study, the strains of *F. falciforme* and *F. solani* clustered closely, supporting the above argument. However, contradicting the nomenclature of the *Fusarium* species continues to be a challenge, resulting in the lack of congruity between morphological and molecular phylogeny. For example, Sang et al. [11] reported *F. cuneirostrum* as a causal agent of FRR in common bean in Uganda, yet these strains were initially identified as *F. solani* f. sp. *Phaseoli* by Munkankusi [47], based on colony characteristics. Ji-wen et al. [48] also reported *F. equiseti* as a member of the *F. incarnatum-equiseti* species complex.

One strain, MitF-487-2, identified as *Clonostachys rhizophaga*, was obtained from LVC agroecology. It was detected by ITS and β tubulin, while TEF1-α did not detect it. This is the first report of *C. rhizophaga* causing wilts in common bean in Uganda. The pathogen is reported to be pathogenic to several crops. It reportedly causes wilts and root rot in chickpeas [49,50,51]. In water chestnut, *C. rhizophaga* causes longitudinal chlorotic streaks and black spots on the stem surface and vascular necrosis [52]. However, some other *Clonostachys* species, such as *C. rosea*, are reported to be mycoparasitic. They are aggressive parasites of fungi, and research on their use for plant disease control is ongoing [53].

Secondary data on DSI were obtained from Erima et al. [18]. All the species differed significantly in DSI caused on common bean. Strains identified as *F. oxysporum* caused more disease than *F. solani*. However, in an earlier study, Chehri et al. [45] observed that *F. solani* caused more disease than *F. oxysporum* on potato tubers. Differences in DSI among different *Fusarium* species were equally observed by Siddique et al. [54] in common bean and by Burlakoti et al. [55] in sugar beet, where *F. graminearum* strains were more pathogenic than *F. oxysporum* strains. The variation in virulence of a single *Fusarium* species in many different crops is an indicator that these species have their primary host on which they proliferate most. The colony colorations of the different species strains in the current study are also related to what other researchers observed. For example, Trebelsi et al. [39] reported the purple coloration of *Fusarium oxysporum*, causing olive trees die back. Tuiime [33] also reported white and brown coloration in *Fusarium solani* fsp *phaseoli.* All the *F. equiseti* isolates in this study had abundant white mycelia, and similar findings were reported by Mohamed et al. [32]. Similarly, the white colony coloration in *F. falciforme* was reported by Dong-Xia [38].

The *Fusarium* species strains in the current study were obtained from different agroecological zones of Uganda. This shows that *Fusarium* species can survive in a wide range of temperatures ranging from the cool humid South Western highlands to warm and less humid North Eastern Dryland. Tusiime [33] reported *F. solani* fsp *phaseoli* in the cool humid regions of the South-Western Highlands. However, in the current study, *F. solani* was reported in various agroecological zones, including in the Northern Mixed farming system, Western Mixed farming system, Lake Victoria crescent and Mbale farmlands, South-Western Highlands and the North-Eastern dry land, which is generally warmer and less humid.

## 5. Conclusions

*Fusarium* species causing root rots and wilts in common beans in Uganda exhibit morphological, phenotypic and genetic diversity. This research has generated information on the diversity of *Fusarium* species causing common bean root rot. The diversity of *Fusarium* species observed in our study needs to be taken into consideration when developing new varieties of breeding programs for the management of the disease. The strains of the different species that have been identified in this study need to be included during germplasm screening so that durable resistance to FRR can be achieved in the released varieties.

## Figures and Tables

**Table 1:** *Fusarium* species strains and accession numbers in the NCBI database. Gene regions that were amplified have accession numbers, while those that failed to amplify do not have accession numbers.

**S/No**	**Strains**	**Agroecology**	**Species**	**Accession Numbers**		
				**TEF1-α**	**Β Tubulin**	**ITS**
1	MbrF-119	WMFS	*F. fabacearum*	PQ497180	PQ497177	PQ363745
2	NakF-106-2	NEDL	*F. oxysporum*	PQ497191	PQ497142	PQ363764
3	KabF-103	SWH	*F. oxysporum*	PQ497198	PQ497143	PQ363757
4	SheF-250-1	WMFS	*F. oxysporum*	PQ497207	PQ497144	PQ363766
5	GomF-492	LVC	*F. oxysporum*	PQ497213	PQ497145	PQ363773
6	KabF-108-1	SWH	*F. oxysporum*	PQ497226	PQ497146	PQ363790
7	KamF-290-2	WMFS	*F. oxysporum*	PQ497234	PQ497147	PQ363797
8	LweF-507	LVC	*F. oxysporum*	-	PQ497148	PQ363804
9	MubF-442-2	LVC	*F. oxysporum*	PQ497182	PQ497148	-
10	MitF-489	LVC	*F. oxysporum*	PQ497181	PQ497150	PQ363746
11	AmuF-513-3	NEDL	F. falciforme	PQ497183	PQ497151	-
12	MubF-463	LVC	*F. oxysporum*	PQ497184	PQ497152	PQ363747
13	KabF-114	SWH	*F. solani*	PQ497185	PQ497153	-
14	GulF-451-1	NMFS	*F. solani*	PQ497186	PQ497154	-
15	SheF-249	WMFS	*F. oxysporum*	PQ497187	PQ497155	PQ363748
16	KolF-563	NMFS	*F. brachygibbosum*	PQ497188	PQ497138	PQ363749
17	LweF-504	LVC	*F. oxysporum*	PQ497189	PQ497176	PQ363750
18	MubF-463-1	LVC	*F. oxysporum*	PQ497190	PQ497156	PQ363751
19	KabF-109-1	SWH	*F. delphinoides*	PQ497192	-	PQ363752
20	OyaF-541-3	NMFS	*F. falciforme*	PQ497193	-	-
21	MubF-234	LVC	*F. oxysporum*	PQ497194	PQ497175	PQ363753
22	KolF-557-4	NMFS	*F. equiseti*	PQ497195	PQ497174	PQ363756
23	LweF-497	LVC	*F. oxysporum*	PQ497196	PQ497173	-
24	ApaF-548	NMFS	*F. solani*	-	PQ497172	PQ363755
25	MitF-491-1	LVC	*F. falciforme*	PQ497197	PQ497171	PQ363791
26	NakF-521	NEDL	*F. equiseti*	PQ497205	PQ497164	PQ363765
27	MubF-462-2	LVC	*F. oxysporum*	PQ497199	PQ497170	PQ363758
28	ApaF-560	NMFS	*F. subflagellisporum*	PQ497200	PQ497169	PQ363759
29	NakF-520	NEDL	*F. equiseti*	PQ497201	PQ497168	PQ363760
30	MubF-465	LVC	*F. oxysporum*	PQ497202	PQ497167	PQ363761
31	ApaF-551	NEDL	*F. equiseti*	PQ497203	PQ497166	PQ363762
32	LirF-602-2	NEDL	*F. equiseti*	-	-	PQ363763
33	NakF-106	NEDL	*F. solani*	PQ497204	PQ497165	PQ363764
34	KyeF-323	WMFS	*F. solani*	PQ497206	PQ497163	-
35	LweF-223	LVC	*F. equiseti*	PQ497208	-	-
36	KapF-372	EH	*F. oxysporum*	-	PQ497162	PQ363767
37	SirF-349-1	LVC	*F. oxysporum*	PQ497209	PQ497161	-
38	KamF-289	WMFS	*F. solani*	-	-	PQ363768
39	IbaF-270	WMFS	*F. oxysporum*	PQ497210	PQ497160	PQ363769
40	ApaF-546	NMFS	*F. equiseti*	-	PQ497159	PQ363770
41	LweF-393	LVC	*F. oxysporum*	PQ497211	PQ497158	PQ363771
42	SirF-358	LVC	*F. falciforme*	PQ497212	PQ497157	PQ363772
43	KabF-113-2	SWH	*F. fabacearum*	PQ497214	PQ497141	PQ363774
44	BusF-258	WMFS	*F. oxysporum*	-	PQ497140	PQ363775
45	MitF-490	LVC	*F. commune*	PQ497215	PQ497139	PQ363776
46	AmuF-518-2	NEDL	*F. equiseti*	PQ497216	PQ497137	PQ363777
47	KamF-493-3	WMFS	*F. oxysporum*	PQ497217	PQ497136	PQ363778
48	KirF-416	WMFS	*F. solani*	PQ497218	PQ497135	PQ363779
49	SirF-358-1	LVC	*F. falciforme*	PQ497219	PQ497134	PQ363780
50	NakF-102-2	NEDL	*F. solani*	-	-	PQ363781
51	KabF-91-1	SWH	*F. oxysporum*	-	-	PQ363782
52	BusF-255-1	WMFS	*F. oxysporum*	PQ497220	PQ497120	PQ363783
53	MubF-464-2	LVC	*F. oxysporum*	PQ497221	PQ497133	PQ363784
54	LweF-296	LVC	*F. commune*	PQ497222	PQ497119	PQ363785
55	NakF-520-1	NEDL	*F. serpentimum*	PQ497223	-	PQ363786
56	MbarF-229	WMFS	*F. commune*	PQ497224	-	PQ363787
57	ApaF-560-1	NMFS	*F. subflagellisporum*	PQ497200	PQ497121	-
58	NakF-105-1	NEDL	*F. oxysporum*	PQ497227	-	-
59	MitF-487-2	LVC	*C. rhizophaga*	PQ363792	-	PQ363792
60	MitF-481	LVC	*F. commune*	PQ497225	PQ497132	PQ363789
61	KyeF-320-2	WMFS	*F. solani*	PQ497227	PQ497131	-
62	ApaF-548-2	NMFS	*F. solani*	PQ497228	-	-
63	SheF-250	WMFS	*F. oxysporum*	PQ497207	-	-
64	MasF-403	WMFS	*F. fredkrugeri*	PQ497229	-	-
65	MitF-491-2	LVC	*F. falciforme*	PQ497230	PQ497171	PQ363788
66	KirF-418	WMFS	*F. falciforme*	PQ497231	PQ497128	PQ363793
67	HoiF-385	WMFS	*F. oxysporum*	PQ497232	PQ497127	PQ363794
68	SirF-349-3	LVC	*F. oxysporum*	PQ497233	PQ497126	PQ363795
69	MitF-487	LVC	*F. oxysporum*	-	PQ497129	PQ363788
70	MubF-466	LVC	*F. oxysporum*	-	-	PQ363798
71	ApaF-546	NMFS	*F. equiseti*	-	PQ497159	-
72	KolF-562	NMFS	*F. solani*	PQ497236	PQ497124	PQ363799
73	KamF-290	WMFS	*F. oxysporum*	-	-	PQ363800
74	LweF-496	LVC	*F. oxysporum*	-	-	PQ363801
75	KolF-562-1	NMFS	*F. solani*	-	PQ497125	-
76	NakF-375	NEDL	*F. oxysporum*	PQ497237	PQ497123	PQ363802
77	Apaf-551-1	NMFS	*F. brevicaudatum*	PQ497233	-	-
78	LweF-215	LVC	*F. oxysporum*	PQ497179	PQ497122	PQ363805
79	ApaF-560	NMFS	*F. oxysporum*	PQ497178	PQ497121	-
80	HoiF-385-1	WMFS	*F. solani*	PQ497219	-	PQ363803

**Table 2:** Average disease severity index (DSI), growth rate and microscopic structures of different *Fusarium* species from Ugandan agroecology zones.

S/No	Organism Name	No. of Strains	* DSI (%)	* Growth Rate (cm/Day)	Microscopic Structues at ×40 Magnification
1	*F. delphinoides*	1	46.8 ± 6.9	0.96 ± 0.01	Rod-shaped nonseptate macro-conidia about 5 to 50 µm lond and spherical micro-conidia
2	*F. solani*	13	36.3 ± 5.9	0.79 ± 0.03	Sickle shaped nonseptate macro-conidia about 5 to 50 µm long.
3	*F. oxysporum*	37	44.4 ± 4.9	0.79 ± 0.03	Rod-shaped septate micro-conidia about 20 to 50 µm long
4	*F. equiseti*	9	47.3 ± 5.9	0.7 ± 0.03	Rod-shaped nonseptate macro-conidia about 50 to 100 µm long. Spherical micro-conidia 2 to 10 µm long
5	*C. rhizophaga*	1	31.3 ± 5.6	0.37 ± 0.05	Oval and Rod-shaped macro-conidia about 10 to 50 µm long
6	*F. subflagellisporum*	2	66.6 ± 10.3	1.2 ± 0	Sperical micro-conidia about 2 to 5 µm long. No macro-conidia
7	*F. fabacearum*	2	40.24 ± 6.0	0.7 ± 0.01	Rod-shaped nonseptate macro-conidia about 5 to 50 µm long. Spheical micro-conidia
8	*F. falciforme*	8	32.3 ± 5.8	0.74 ± 0.03	Rod-shaped macro-conidia about 50 to 150 µm long. Spherical micro-conidia
9	*F. brachygibbosum*	1	65.8 ± 5.7	0.87 ± 0.03	Oval nonseptate macro-conidia about 10 to 30 µm long
10	*F. brevicaudatum*	1	59.3 ± 4	0.6 ± 0.01	Isolates in storage failed to regenerate for microscopy
11	*F. commune*	4	62.5 ± 6.0	0.88 ± 0.03	Rod-shaped nonsepate macro-conidia about 5 to 50 µm,
12	*F. serpentimum*	1	45.1 ± 6.1	0.17 ± 0.02	Isolates in storage failed to regenerate for microscopy
13	*F. frekrugeri*	1	40.3 ± 4.1	0.87 ± 0.03	Oval nonseptate macro-conidia up to about 40 µm long. Spherical micro-conidia 5 to 10 µm

* Disease severity index (DSI) and growth rate data were obtained from Erima et al. [18].

## Data Availability

The partial sequences of TEF 1α, βtubulin and ITS can be obtained from the NCBI database under ID; 288260, 288276 and 2873058 for TEF 1α, β tubulin and ITS, respectively. The rest of the data are within the article.

## Supplementary Materials

The following supporting information can be downloaded at: https://www.mdpi.com/article/10.3390/jof11040283/s1, Figure S1: Colony morphology of 10 day old *Fusarium* species strains on PDA. A- Morphology on the top of the Petri dish and B- Morphology on the bottom of the Petri dish; Table S1: Colony colour, growth rate and virulence of *Fusarium* spp. strains following inoculation on five common bean varieties in the screenhouse. artial sequences of Fusarium species strains can be obtained at NCBI under ID#2884260, 2883276, 2873058 for TEF-1α, β tubulin and ITS respectively.
