# Supplementary material for: Molecular Characterisation of Fusarium Species Causing Common Bean Root Rot in Uganda
Source: J Fungi (Basel). 2025 Apr 3;11(4):283. doi: 10.3390/jof11040283 (PMC12028566; doi:10.3390/jof11040283)
Supplement: Supplementary file 1 [file jof-11-00283-s001.zip › Figure S1.pdf]

Figure S1: Colony morphology of 10 day old *Fusarium* species strains on PDA. A- Morphology on the top of the Petri dish and B- Morphology on the bottom of the Petri dish

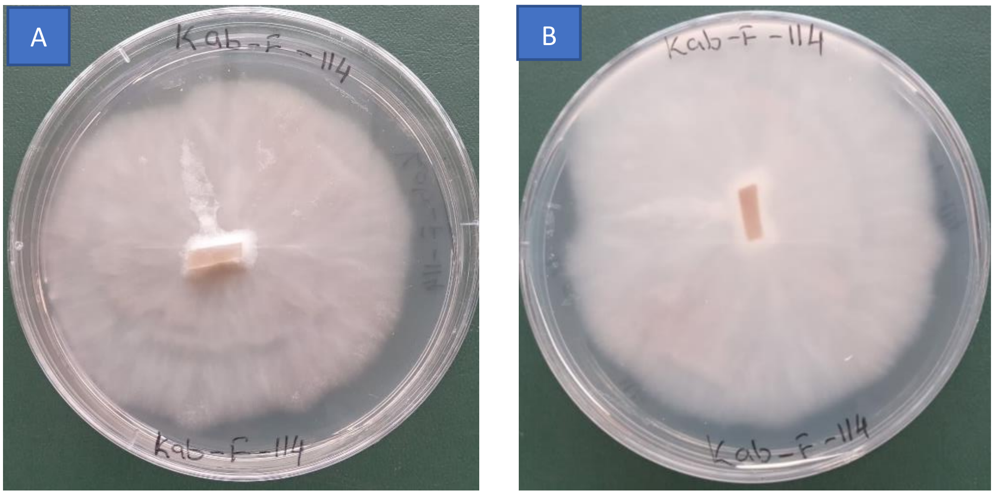

(a) Strains KABF-114- *F. solani*

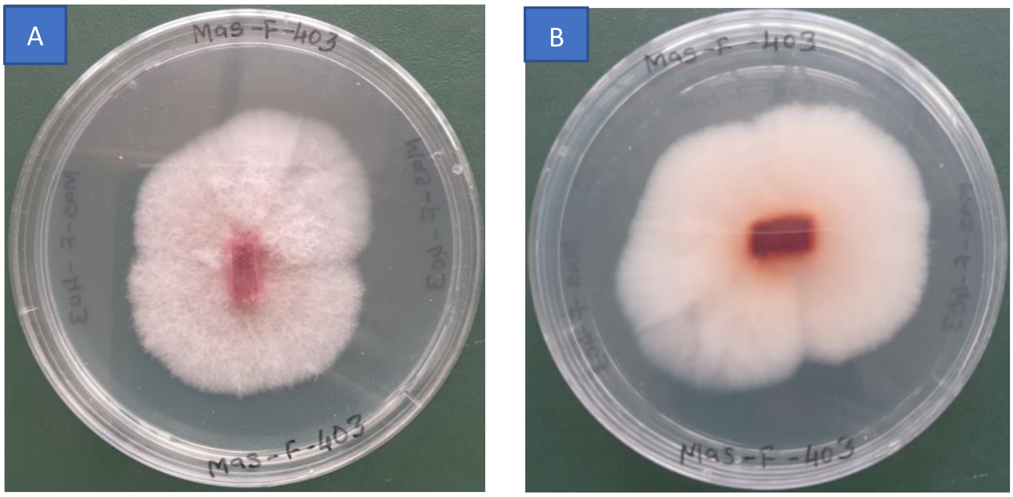

(b) Strain MASF-403- *F. frekrugeri*

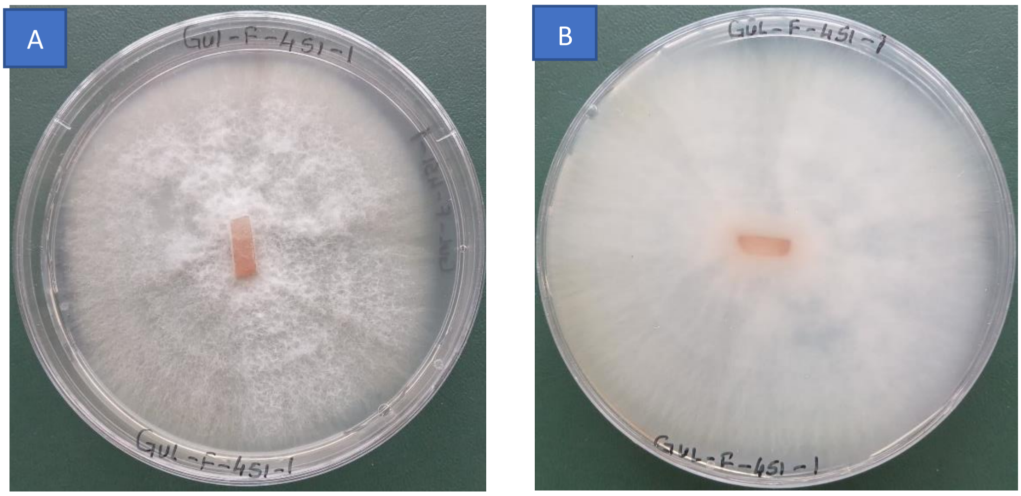

(c) Strain GULF-451-1- *F. solani*

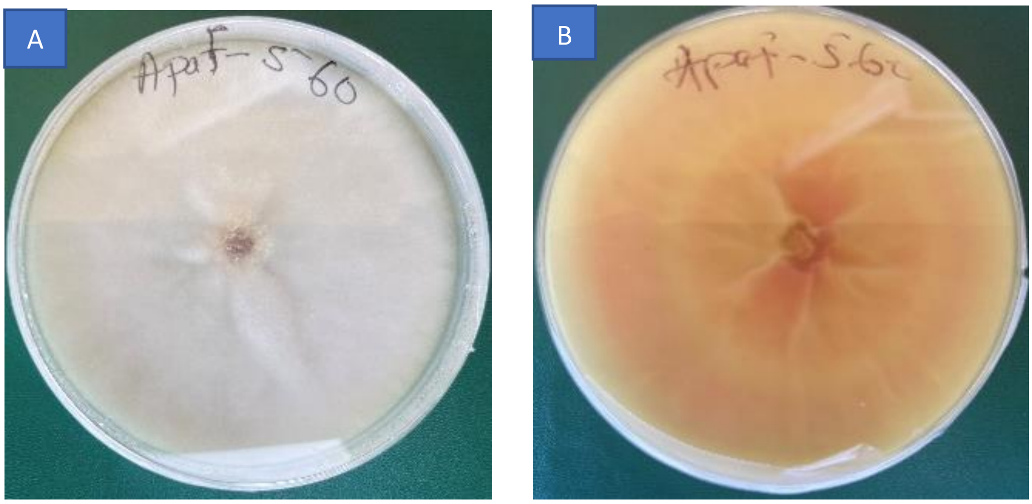

(d) Strain APAF-560- *F. subflagellisporum*

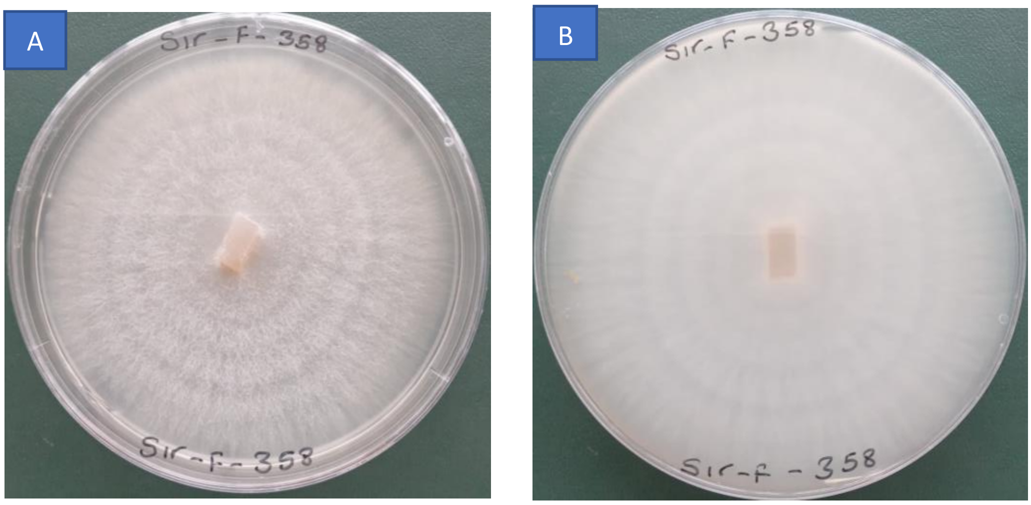

(e) Strain SIRF-358- *F. falciforme*

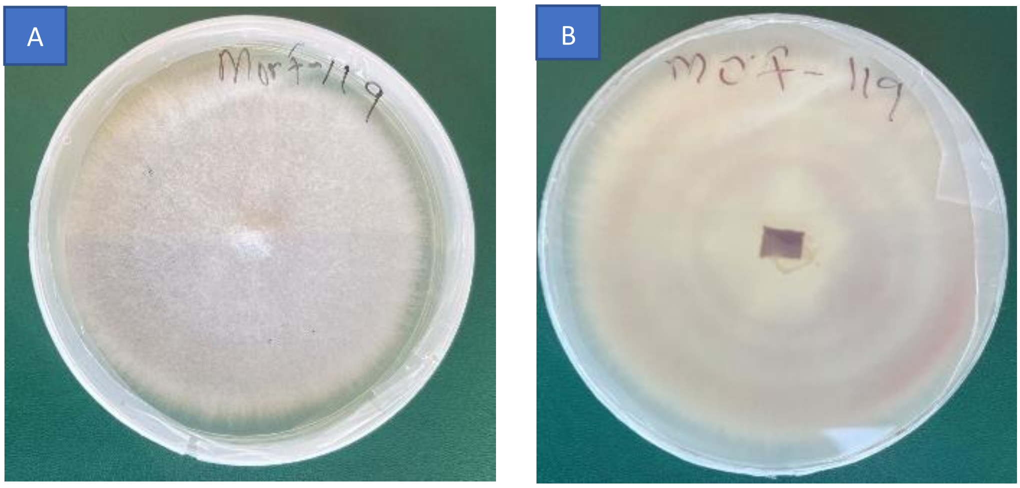

(f) Strain MORF-119- *F. fabacearum*

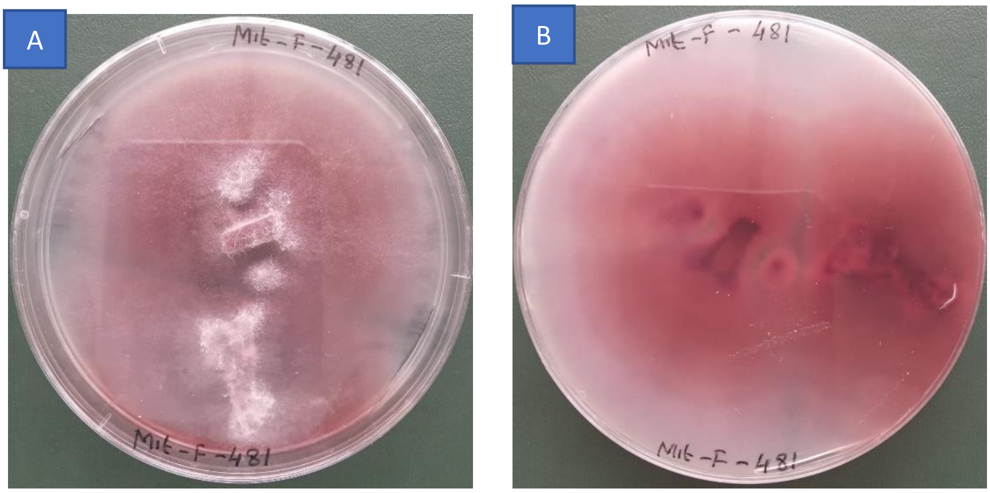

(g) Strain MITF-481-*F. commune*

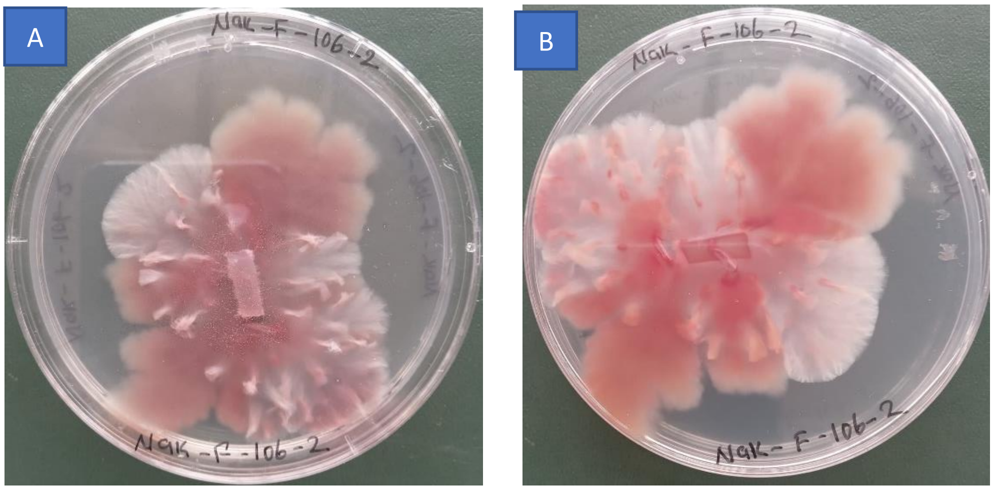

(h) Strain NakF-106-2- *F. oxysporum*

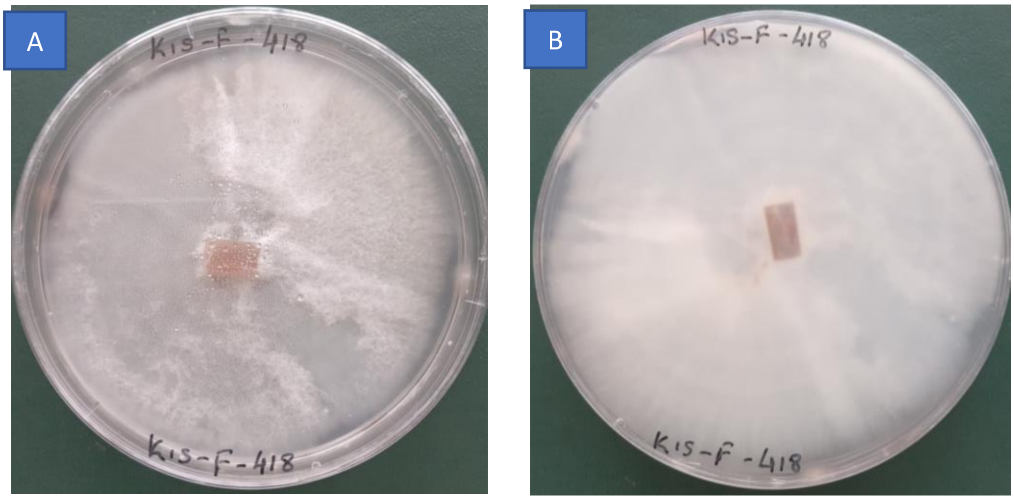

(i) Strain KIRF-418-*F. falciforme*

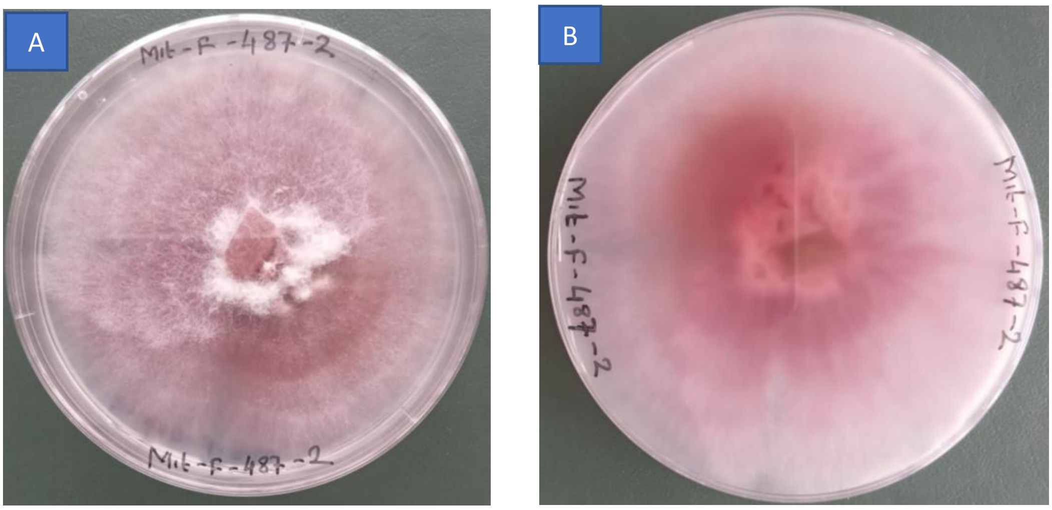

(j)Strain MITF-487-2- *C. rhizophaga*

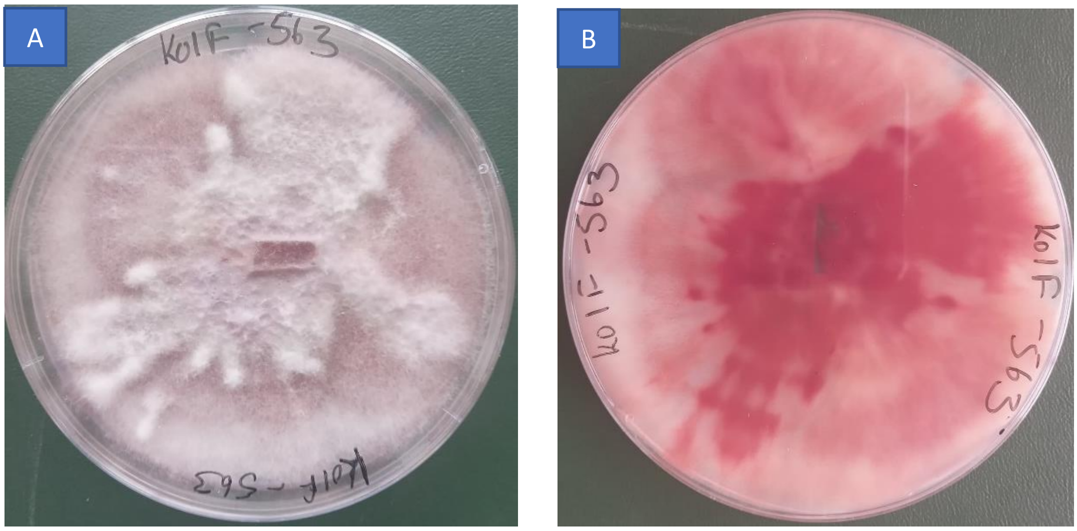

(k) Strain KOLF-563- *F. brachygibbosum*

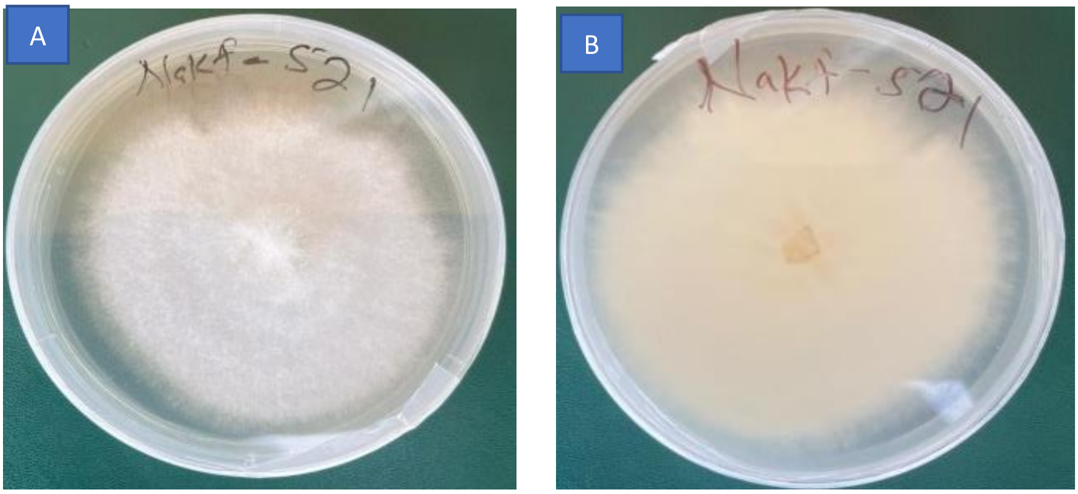

(l) Strain NAKF-521- *F. equiseti*

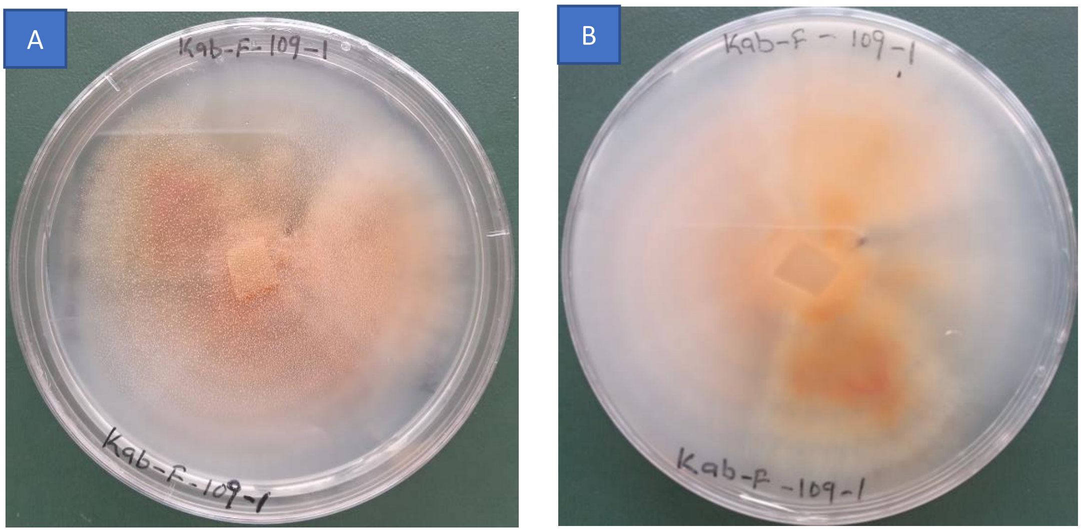

(m) Strain KABF-109-1-*F. delphinoides*

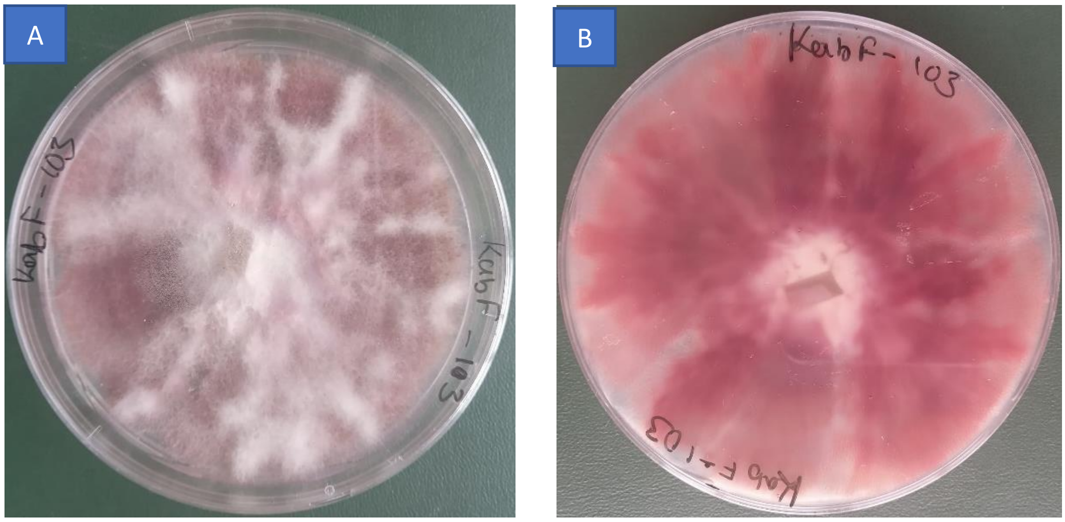

(n) Strain KABF-103-*F. oxysporum*
