# Supplementary material for: Molecular Characterisation of Fusarium Species Causing Common Bean Root Rot in Uganda
Source: J Fungi (Basel). 2025 Apr 3;11(4):283. doi: 10.3390/jof11040283 (PMC12028566; doi:10.3390/jof11040283)
Supplement: Supplementary file 1 [file jof-11-00283-s001.zip › Table S1.pdf]

Table S1. Colony colour, growth rate and Pathogenicity of *Fusarium* spp. strains following inoculation on five common bean varieties in the screenhouse

S/no.	Strain	*Fusarium* species	Colony colour on media	Growth rate (cm/day)	SE	DSI (%)	SE
1	AMUF-513-3	*F. falciforme*	White	0.98	0.01	26.4	4.1
2	APAF-548-2	*F. solani*	White/cream	0.73	0.04	16.2	3.3
3	AMUF-518-2	*F. equiseti*	White	0.90	0.01	63.3	4.2
4	APAF-546	*F. equiseti*	White	0.94	0.15	42.2	2.6
5	APAF-548	*F. solani*	White/cream	0.91	0.02	54.8	3.0
6	APAF-551	*F. equiseti*	White	0.6	0.12	59.3	7.8
7	APAF-560-1	*F. subflagellisporum*	Pink/Brown	1.1	0	60.4	10.0
8	APAF-560	*F. subflagellisporum*	Pink/Brown	1.2	0	66.4	10.7
9	BUSF-258	*F. oxysporum*	White/Pink	0.91	0.03	61.4	4.8
10	BUSF-255-1	*F. oxysporum*	White/Orange	0.69	0.02	22.0	3.6
11	BUSF-258-1	*F. oxysporum*	White/Pink	0.96	0.02	87.0	7.5
12	GOMF-492	*F. oxysporum*	White/Pink	0.73	0.03	15.0	2.3
13	GULF-451-1	*F. oxysporum*	White/pink	0.76	0.02	8.1	7
14	HOIF-385	*F. oxysporum*	White/Purple	0.76	0	0.9	0.9
15	IBAF-270	*F. oxysporum*	White/Pink	0.92	0.05	39.1	5
16	KABF-113-2	*F. fabacearum*	White	0.55	0.01	35.9	8.6
17	KABF-114	*F. solani*	White/Orange	0.77	0.04	35.2	6.3
18	KABF-91-1	*F. oxysporum*	White	0.88	0.02	28.8	6
19	KABF-103	*F. oxysporum*	White/purple	0.93	0.04	98.3	1.6
20	KABF-108-1	*F. oxysporum*	White	0.86	0.01	63.2	10.8
21	KABF-109-1	*F. delphinoides*	White/Pink	0.87	0.01	62.8	6.9
22	KAMF-290	*F. oxysporum*	White	0.84	0.01	34.5	5.9
23	KAMF-290-1	*F. oxysporum*	White	0.9	0.03	48.7	5.6
23	KAMF-493-3	*F. oxysporum*	White	0.8	0.02	55.6	5.9
24	KAMF-289	*F. solani*	White	0.72	0.04	22.0	6.1
25	KAPF-372	*F. oxysporum*	White/Cream	0.22	0.12	50.0	4.2
26	NakF-102-2	*F. solani*	White/Purple	0.94	0.04	63.5	4
27	KIRF-418	*F. falciforme*	White	0.8	0.01	30.0	3.4
28	KIRF-416	*F. solani*	White/Yellow	0.75	0.02	14.0	6.5
29	KOLF-557-4	*F. equiseti*	White	1.07	0.02	61.8	10.6
30	KOLF-562-1	*F. solani*	White/Pink	0.97	0.01	59.6	8
31	KOLF-562	*F. solani*	White/Pink	1.06	0.02	72.6	5
32	KOLF-563	*F. brachygibbosum*	White/Purple	0.87	0.03	65.8	5.7
33	KYEF-320-2	*F. solani*	White	0.76	0.03	49.4	9
34	KYEF-323	*F. solani*	White	0.89	0.05	46.3	6.3

S/no.	Strain	*Fusarium* species	Colony colour on media	Growth rate (cm/day)	SE	DSI (%)	SE
35	LIRF-601-2	*F. equiseti*	White	0.87	0.09	23.4	5
36	LWEF-215	*F. oxyspsorum*	White	0.67	0.03	48.2	4
37	LWEF-223	*F. equiseti*	White	0.65	0.17	70.0	5.3
38	LWEF-296	*F. commune*	White/Pink	0.92	0.01	51.0	6.1
39	LWEF-497	*F. oxysporum*	White/Purple	0.85	0.09	30.5	4
40	LWEF-496	*F. oxysporum*	White	0.91	0.01	50.3	4.5
41	LWEF-504	*F. oxysporum*	White	0.59	0.04	32.6	5.3
42	LWEF-507	*F. oxysporum*	White/Purple	0.86	0.08	55.1	8.1
43	LWEF-604	*F. equiseti*	White	0.75	0.05	58.1	5.3
44	LWEF-215	*F. oxysporum*	White/Purple	0.57	0.01	19.3	7.8
45	LWEF-497	*F. oxysporum*	White	0.63	0.09	23.7	3.5
46	MASF-403	*F. fredkrugeri*	White	0.87	0.03	41.0	4.1
47	MBARF-229	*F. commune*	White/Purple	0.81	0.03	37.0	7.2
48	MITF-487	*F. oxysporum*	White	0.85	0.04	31.3	5.6
49	MITF-489	*F. oxysporum*	White	0.55	0.03	42.4	5.3
50	MITF-489-2	*F. falciforme*	Cream/White	0.64	0.03	42.8	3.4
51	MITF-491	*F. falciforme*	White/Purple	0.69	0.03	52.6	4
52	MITF-481	*F. commune*	White/Purple	0.82	0.03	73.1	8.8
53	MITF-490	*F. commune*	White	1.03	0.04	88.3	5
54	MITF-491-1	*F. falciforme*	White/Purple	0.69	0.03	27.8	4
55	MITF-487-1	*F. oxysporum*	White	0.85	0.02	75.0	3
56	MORF-119	*F. fabacearum*	Cream	1.07	0.01	57.4	5.7
57	MUBF-463	*F. oxysporum*	White	0.86	0.03	60.7	8
58	MUBF-465	*F. oxysporum*	White/Purple	0.87	0.05	30.1	5.8
59	MUBF-466	*F. oxysporum*	White/Purple	0.52	0.02	61.0	11
60	APAF-551-1	*F. brevicaudatum*	White	0.6	0.01	59.3	4
61	MUBF-461	*F. oxysporum*	White/Pink	0.78	0.04	62.0	10
62	MUBF-462-2	*F. oxysporum*	White/Purple	0.71	0.02	21.9	5.1
63	NAKF-105-1	*F. oxysporum*	White	0.6	0.03	46.0	5.1
64	NAKF-106	*F. solani*	White/Purple	0.31	0.01	40.1	5.1
65	NAKF-106-2	*F. oxysporum*	White	0.69	0.03	28.3	3
66	NAKF-375	*F. oxysporum*	White	0.68	0.03	57.3	11.6
67	NAKF-520	*F. equiseti*	White	0.17	0.02	45.1	4.9
68	NAKF-520-2	*F. serpentimum*	White/Purple	0.54	0.02	42.3	6.1
69	NAKF-521	*F. equiseti*	White	1.21	0.02	58.9	8.9
70	NAKF-102	*F. solani*	White/Purple	0.79	0.03	20.8	3
71	HOIF-385-1	*F. solani*	White	0.31	0.02	39.5	4
72	OYAF-541-2	*F. falciforme*	White	0.63	0.08	34.9	5
73	OYAF-541-3	*F. falciforme*	White/Purple	1.06	0.02	76.0	6.6

S/no.	Strain	*Fusarium* species	Colony colour on media	Growth rate (cm/day)	SE	DSI (%)	SE
74	SHEF-249	*F. oxysporum*	White/Purple	0.88	0.04	46.2	3.2
75	SHEF-250	*F. oxysporum*	White	0.9	0.03	30.2	9.8
76	SHEF-250-1	*F. oxysporum*	White/Purple	0.64	0.09	18.2	10
77	SIRF-349-1	*F. oxysporum*	White	0.93	0.02	59.0	10.9
78	SIRF-349-3	*F. oxysporum*	White/Pink	0.83	0.02	73.8	7.8
79	SIRF-358	*F. falciforme*	White	0.75	0.06	25.7	4.1
80	MITF-487-2	*C. rhizophaga*	White/Purple	0.37	0.05	31.4	5.6

[1]EH = Eastern Highlands, LVC = Lake Victoria Crescent and Mbale Farmlands, KP = Karamoja Pastoral zone, NMFS = Northern Mixed Farming System zone, SWH = Southwestern Highlands, WMFS = Western Mixed Farming System zone.

[2]Mean disease severity index (DSI) caused by each strain on the five varieties combined.
